# Dual‐Wavelength Control of Charge Accumulation in Rubrene Microcrystals with Anisotropic Conductivity

**DOI:** 10.1002/advs.76246

**Published:** 2026-06-23

**Authors:** Moha Naeimi, Ingo Barke, Sylvia Speller

**Affiliations:** ^1^ Institute of physics University of Rostock Rostock Germany; ^2^ Department of light, life and matter University of Rostock Rostock Germany

**Keywords:** Charge, Exciton, PEEM, Rubrene

## Abstract

Previously, a novel type of rubrene microcrystals was reported, forming two distinct sectors–diamond‐ and triangular‐shaped–that exhibit pronounced contrasts in photoluminescence (PL) spectra and exciton dynamics. In the present work, their internal electronic structure is investigated using time‐of‐flight photoemission electron spectroscopy (TOF‐PES), revealing that the two sector's different charging characteristics arise from anisotropic conductivities. Upon photoemission via a one‐photon photoemission (1PPE) process excited by 6.2 eV (200 nm) photons, the diamond‐shaped sectors accumulate significant charge, whereas the triangular sectors remain essentially uncharged. The charge accumulation in the diamond sectors can be neutralized by additional sub‐threshold illumination, which generates charge carriers through internal photoeffect. The dynamics and energetics of the observed band shifting is described quantitatively by a model combining surface capacitance and drift‐diffusion. These crystalline systems enable the creation of built‐in charge landscapes that can be manipulated both spatially and temporally.

## Graphical abstract

Rubrene microcrystals, forming two distinct sector types, are investigated using photoemission electron spectroscopy. The two sectors exhibit a built‐in charge landscape, arising from anisotropic conductivities. Upon excitation with UV light, the diamond‐shaped sectors accumulate significant charge, whereas the triangular sectors remain essentially uncharged. Charge accumulation can be neutralized by additional sub‐threshold illumination, generating charge carriers through internal photoeffect.

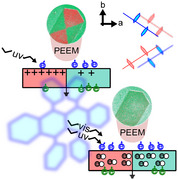

## 1 Introduction

Organic crystals and aggregates have gained significant attention in organic electronics and materials science due to their unique electronic, optical, and structural properties. With a tetracene backbone and 4 phenyl groups, rubrene is subject to the huge interest of research with numerous applications in electronic devices ranging from field effect transistors [1, 2] to light emitting diodes [3], offering one of the highest charge mobilities [4] among organic molecules and a very efficient singlet fission in the crystalline form. Through singlet fission [5, 6, 7] at ∼530 nm, long‐lived triplet excitons are generated, which can diffuse extensively within the bulk, as well as along the surface, playing a crucial role in energy transport and interfacial processes [8]. Furthermore, its strong absorption in the visible range and favorable energy level alignment with common acceptor materials have driven extensive research into rubrene‐based solar cells [9, 10]. Despite these advantages, the photovoltaic performance of rubrene remains highly dependent on crystal quality, morphology, and interface engineering, highlighting the need for controlled growth strategies to fully exploit its potential in next‐generation organic photovoltaics.

For efficient charge and exciton transfer, the morphology of the molecular and lattice structure plays a critical role. Previous studies have shown that this process is strongly suppressed in the amorphous phase of rubrene [11, 12]. The remaining challenge, therefore, lies in preparing large, single‐domain, and flat crystals in the orthorhombic phase [13], where the singlet fission is most efficient [14, 15]. Various strategies may be employed to address this challenge, including thermal treatment [16], and crystal growth under extreme conditions such as physical vapour transport (PVT) [17] or high partial pressure [18].

We recently reported a novel type of rubrene orthorhombic crystals [19] growing under high partial pressure and elevated temperature. These crystals tend to have either the **c**‐ or **b**‐axis of the orthorhombic unit cell as the out‐of‐plane axis. The out‐of‐plane axes significantly impact the PL spectra and exciton dynamics arising from the orientation of the rubrene transition dipole moment, which is aligned along the **c**‐axis of the unit cell. In both growth directions, a particular growth habit was observed exhibiting four sectors of two distinct types within one single crystal, determining PL spectra and exciton dynamics. The source of this zone‐sectoring is the rotation of the orthorhombic unit cell around axes during the growth impacting the absorption rates and exciton diffusion direction along the crystal in‐ and out‐of‐ plane.

Understanding the fundamental electronic structure of rubrene single crystals is essential for investigating their charge transport and photo‐voltaic properties. Angle‐resolved photoelectron spectroscopy (ARPES) experiments showed that the highest occupied molecular orbital (HOMO) valence band in rubrene exhibits a dispersion of 0.4 eV along the strongly π−π‐stacked crystallographic directions (**a**‐axis in orthorhombic phase), supporting a band‐like transport in rubrene [20]. Subsequent wavelength dependent photoemission studies further resolved the 2D valence band structure, revealing anisotropy in intermolecular transfer confirming the quasi‐2D nature of the HOMO band [21]. More recent angle‐resolved measurements have shown that near‐surface electronic doping via molecular electron donors and acceptors can shift the Fermi level within the bandgap of rubrene without significantly perturbing the intrinsic valence band dispersion, indicating that charge injection and accumulation processes can be probed [22].

We present a photoemission study on the zone‐sectored rubrene microcrystal, showing that the diamond‐shaped sectors accumulate significant charge under a one‐photon photoemission (1PPE) process when excited with photons above the work function, while the triangular sectors remain largely unaffected. Analysis of the charge dynamics indicates that the sectors exhibit strongly anisotropic conductivities and behave like micro‐scale capacitors, characterized by a distinct time constant. The photoconductivity and subsequent charge could be controlled with another excitation with lower photon energies by generating electron‐hole pairs resulting from internal photo‐effect.

## 2 Experimental Methods

The preparation method of the crystals is described in our previous work [19]. For surface potential investigations, we used an atomic force microscope (Park Systems NX20) in sideband Kelvin probe mode with conductive tips coated by a chromium platinum (Cr‐Pt) layer exhibiting a cantilever spring constant of 3 N/m and a free eigen frequency of 75 kHz.

The time‐of‐flight photoemission electron spectroscopy was conducted in a PEEM (Focus IS‐PEEM) by placing the sample in a vacuum chamber with a base pressure of $10^{-10}$ mbar. The light angle of incidence was 23 degrees grazing, ensuring a good alignment of the light polarisation and transition dipole moment of **c**‐oriented rubrene crystals, which is perpendicular to the substrate.

We used two different light sources for photoemission and surface potential investigations: (1) The 4th harmonics of a tunable Ti:Sa femtosecond (fs) laser (Mira 900F) yielding photons with energies of 6.2 eV (200 nm) with the repetition rate of 1 Mhz and pulse duration of 200 ps. (2) A continues wave (cw) laser with photon energies of 3.09 eV (405 nm).

Visualization and analysis was done by Gwyddion [23] and Igor Pro (Wavemetrics). We calculated an apparent binding energy from the kinetic energy of the detected electrons, assuming a one‐photon photoemission process. In case of charging, this “apparent” binding energy will not reflect the mere binding energy but additionally accounts for the reduction in the photo electron–s kinetic energy by the positive charging of the surface.

## 3 Results and Discussion

**FIGURE 1:**
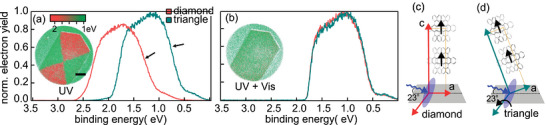
(a) 1PPE electron spectra of a rubrene crystal resolved for different sectors 6.2 eV illumination. (b) 1PPE electron spectra of the same crystal upon two color illumination (6.2 and 3.1 eV). The insets are PEEM maps which color‐code the spatially‐resolved centre of spectral weight. The charge patterns resemble the diamond‐ and triangular‐shaped sectors. (scale bar: 10 μm) (c and d) Schematic of the orthorhombic **ac**‐plane in diamond‐ and triangular‐shaped sectors, respectively. The blue arrows and circles, represent the excitation light and polarization plane, applied with the angle of $23^{\circ }$ grazing. Note that the spectra for the diamond sector are shifted toward higher ”apparent” binding energies.

We investigated a **c**‐oriented rubrene microcrystal (see [19]), in which the crystallographic **c**‐axis i.e., the transition dipole moment of rubrene molecules in the orthorhombic phase is perpendicular to the surface. A key advantage of this crystal orientation is that, under grazing incidence, the polarization plane of the excitation light aligns well with the transition dipole moment, resulting in enhanced absorption if excitons are involved. The distinction between the diamond‐ and triangular‐shaped sectors of this crystal arises from the rotation of the unit cell around the **b**‐axis (see Figure 1c,d). In the triangular sectors, this rotation is more pronounced than in the diamond sectors, as shown in our previous work [19]. Such rotation impacts the photoluminescence spectra and exciton dynamics.

Figure 1a shows the electron spectra of a **c**‐oriented crystal, resolved for different sectors. The excitation source is a 6.2 eV (200 nm) femtosecond pulsed laser (here we refer to it as “UV” light) operating at a repetition rate of 1 MHz with an average power of 2 nW. The electrons are emitted through a 1PPE process. The acquisition time of the spectra is 20 s, while the sample was already exposed to the illumination for more than 5 min. As shown, the spectra from the diamond sectors are shifted by approximately 0.75 eV toward higher binding energies, while the overall spectral shape remains unchanged. The different slopes at the high kinetic energies (marked with arrows in Figure 1a) are mainly caused by a residual time‐dependent shift of the spectrum within the relatively long data acquisition time.

Figure 1b shows the electron spectra when an additional illumination source is simultaneously applied. The second illumination is a 3.06 eV (405 nm) continuous wave laser (here we refer to it as “vis” light) with the same power as the UV source. While no additional photoemission is induced by the vis light, the spectra from different sectors are similar. We attribute the weak peak at lower binding energies to possible trap states caused by crystal defects or to the triplet state (1.14 eV) generated via singlet fission [24]. The width of the band measures about 1 eV. This is wider than ARPES studies [25], however, we tentatively attribute the band to the HOMO band (highest occupied molecular orbital) having in mind our extraction lens collects electrons with all k‐vectors.

As the entire spectra of the diamond sectors shift toward higher binding energies without altering the spectral shape, this behavior can be attributed to charging, which is more pronounced in diamond‐shaped sectors compared to the triangular‐shaped ones. In the photoemission process, charging is the result of hole accumulation within the semiconductor as photoelectrons are liberated. This accumulation of positively charged holes cannot be compensated by electron injection from the substrate and further electron emission, thereby resulting in a shift toward “apparently” higher binding energies [26, 27].

The spectral shifts due to charge, i.e., hole accumulation, is different from spectral shifts due to surface photo voltage [28]. The surface photovoltage (SPV) effect is explained in the band bending picture [29, 30], depending on the doping of semiconductor, i.e. n‐type and p‐type, where the conduction and valance band edges bend upward and downward the surface, respectively, to equilibrate the Fermi level. The SPV then shifts the electron spectra toward higher binding energies in n‐type and lower binding energies in p‐type semiconductors.

### 3.1 RC Charging Model

**FIGURE 2:**
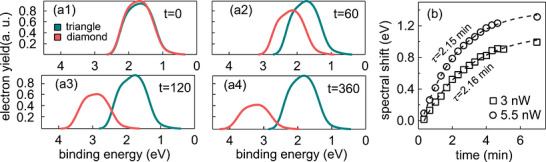
It takes some time for the crystal to fully charge. (a) Electron spectra of the crystal upon illumination (6.16 eV) for different times after illumination is started. (b) Shift of spectra (charge) versus time after illumination has started for two different illumination powers.

As we observed, charging does not occur immediately; instead, it takes time for the crystal sectors to fully charge. Figure 2a shows a series of electron spectra taken at different times after illumination, each for 20 s. Initially, both sectors share the same spectral position. However, after 60 s, the spectrum of the diamond‐shaped sectors shifts by nearly 0.6 eV toward higher binding energies. Over time, the spectral shift continues, while the spectrum of the triangular‐shaped sectors seems to remain unchanged. This process persists until the spectral shift reaches a saturation point. The long characteristic time of few minutes is compatible with bulk anisotropy, not with surface states.

We evaluated the charge dynamics for different UV illumination intensity and observed that higher UV power results in stronger spectral shift and charging. Interestingly, the time constant for the charge to reach the saturation, i.e., “fully charged” is independent of the illumination power. Figure 2b is the shift of spectra versus UV illumination time for two different illumination intensities. Both datasets follow the rate equation:

(1) $$V\left(t\right)=V_{0}+V_{1}\cdot \left(1-e^{-t/\tau }\right)$$

yielding the same time constant of ≈2.15 min. Here, V is the spectral shift in eV and $V_{0}$ and $V_{1}$ are the offset and maximum spectral shift, respectively. The measured time constant varies across different samples. The charge dynamics follow a rate equation, suggesting the presence of an internal or light‐induced resistance that opposes hole accumulation, although the charging process due to hole accumulation remains dominant.

**FIGURE 3:**
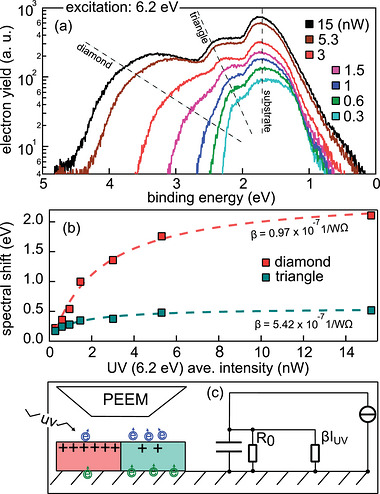
Different crystal domains are charge differently and the more excitation intensity is, the more charge is stored. (a) Overall electron spectra of the crystal for different illumination powers. The dashed lines indicate the sector and substrate specific spectral bands. (b) Spectral shift versus the UV illumination average power, resolved for different sectors. (c) Schematic of single color experiment. left panel: Red and green rectangles represent the diamond‐ and triangular‐shaped sectors. The blue and green circles represent the photoelectrons and electron supplement from the substrate. The positive charge accumulation near the surface is indicated with + sign. Right panel: Schematic of RC model with $R_{0}$ and $\beta I_{UV}$ representing the dark resistance of rubrene crystal and resistance induced by UV illumination, respectively.

Figure 3a shows the overall electron spectra of the crystal, including the diamond‐ and triangular‐shaped sectors. More illumination power leads to more charge of the diamond sector. All of the spectra were taken in a 5‐min data acquisition, well longer than the charging time constant. A slight spectral shift of the triangle sectors is also observed. Figure 3b shows the spectral shift of the crystal versus the illumination intensity, resolved for different sectors.

We describe this spectral shift and charge using the RC model, where hole accumulation due to the UV illumination is considered as the charge stored in the capacitor with capacitance C and resistance R. Figure 3c, is a schematic of the experiment, where the positively charged holes are accumulated differently in different sectors near the surface of the crystal. The diamond‐ and triangular‐shaped sectors are schematically shown as red and green, respectively. Green arrows indicate the electron supplement from the substrate, to be replaced by the emitted electrons, indicated by blue arrows. Figure 3c, also shows the corresponding RC model, where $\frac{1}{R_{0}}$ is the intrinsic conductance of the rubrene crystal and $\beta I_{UV}$ represents the induced conductance due to generated excitons by the UV lights. Although the generated excitons could increase the conductance, the excitons being generated by UV light (6.2 eV) are expected to be short lived. We believe that a few top layers of the crystal is the active length of the capacitor.

Given V=αI·R, with I being the UV illumination intensity and $R=\frac{1}{\frac{1}{R_{0}}+\beta I}$ the equivalent parallel resistance of the crystal dark resistance and the resistance due to electron‐hole generation via UV with rate β, the spectral shift versus the UV illumination intensity could be written as:

(2) $$V\left(I_{UV}\right)=V_{0}+\frac{\alpha R_{0}I_{UV}}{1+\beta R_{0}I_{UV}}$$

$\alpha =e\frac{dr}{dI_{UV}}$ is proportional to the photoemission rate upon UV illumination with r being the photoelectron count rate with the unit of A/W. β has the unit of 1/ΩW and indicates the photoconductivity i.e., how efficiently intensity opens conduction channels. The obtained fitting parameters yields $R_{0}=3.6\times 10^{15}\Omega$ and $R_{0}=1.15\times 10^{15}\Omega$ for diamond‐ and triangular‐shaped sectors, respectively. This explains our observation, in which charge pattern in the crystal remains intact when the illumination is stopped. Accordingly, the photoconductivity factor is obtained $\beta =0.97\times 10^{-7}1/\Omega W$ and $\beta =5.42\times 10^{-7}1/\Omega W$ for diamond‐ and triangular‐shaped sectors.

**FIGURE 4:**
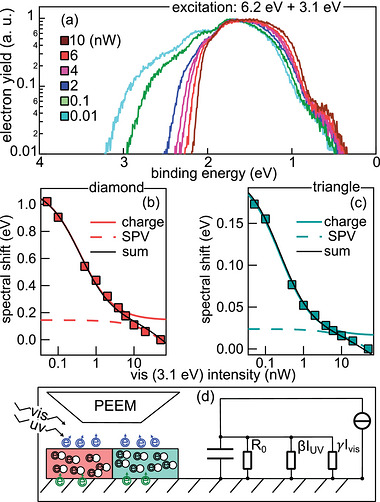
The vis light can suppress the charge induced by the UV light. (a) Electron spectra of rubrene single crystals illuminated simultaneously by UV (1.4 nW) and different vis powers. (b and c) Spectral shift versus vis illumination power, resolved for different sectors. (d) Schematics of double‐color experiment. left panel: Red and green rectangles represent the diamond‐ and triangular‐shaped sectors. The blue and green circles represent the photoelectrons and electron supplement from the substrate. The black and white circles indicate the excitons generated via internal photoelectric upon vis light. right panel: Schematics of corresponding RC model with $R_{0}$ representing the dark resistance, $\beta I_{UV}$ and $\gamma I_{vis}$ being the photo resistance due to UV and vis illumination, respectively.

Once we apply the vis light (3.1 eV) simultaneously with the UV light (6.2 eV), the charge pattern neutralizes, instantaneously. Interestingly; the vis light neutralizes the charge without inducing any further photoemission. Figure 4a shows the overall electron spectra of the crystal with UV–vis illumination, at fixed UV intensity (1.4 nW) and different vis illumination intensities. Each spectra is acquired in 5 min. As shown in Figure 4a, increasing the vis illumination intensity results in the reduction of spectral shift pointing to charge suppression. At I=10nW, the charge seems to be neutralized, leading the spectra from the diamond‐ and triangular‐shaped regions to converge (see also Figure 1b).

Figure 4b,c show the spectral shift versus the vis light intensity for the diamond‐ and triangular‐shaped sectors, respectively. As shown, the spectra from both sectors tend to shift further toward lower binding energies, even after the charge is fully suppressed. Here, we model the situation using two separated approaches. First, for the intensities below ∼ 10 nW, the spectral shift is due to charge suppression as a result of high efficient electron‐hole generation upon vis illumination. This process adds another resistance against the hole accumulation. In the contrary of the UV illumination, in the vis illumination, the exciton generation is rather efficient [24, 31] and the singlet fission is the dominant process leading to long lived triplet excitons. We cannot rule out the role of singlet exciton dissociation in enhanced photoconductivity. The schematics of the RC model is shown in Figure 4d. Figure 4d also schematically represents the charge suppression upon vis illumination. The diamond‐ and triangular‐shaped sectors are represented by red and green, and the generated electron holes are represented by black and white circles. Hence, the spectral shift could be expressed as:

(3) $$V\left(I_{vis}\right)=V_{0}+\frac{\alpha R_{0}I_{UV}}{1+\beta R_{0}I_{UV}}{\left(1+\frac{\gamma R_{0}}{1+\beta R_{0}I_{UV}}I_{vis}\right)}^{-1}$$

With γ being the photoconductivity induced by the vis light. Fitting this equation to the data and using the obtained parameters from the single‐color experiment, yields $\gamma =0.97\times 10^{-6}1/\Omega W$ for diamond‐shaped and $\gamma =4.7\times 10^{-6}1/\Omega W$ for triangle‐shaped sectors. The solid colored curves in Figure 4b,c show the fit. Comparing γ and β, which are photoconductivity factors upon vis and UV illumination, respectively, the obtained values are in line with the fact that the vis light generates electron‐holes more efficiently than the UV light at least one order of magnitude. Figure S6 is a 3D plot showing the charge (spectral shift) of the rubrene sample as a function of UV and vis excitation intensities.

### 3.2 Drift Diffusion Correction

Once the charge in both sectors is neutralized, the spectral shift due to additional increase in the vis light intensity, i.e., intensities of the vis light above ≈10 nW, could be described by the surface photovoltage, determined by the drift diffusion model:

(4) $$V\left(I_{vis}\right)=V_{0}+n\frac{k_{B}T}{e}\ln\left(\frac{I_{vis}}{I_{0}}+1\right),$$

where $\frac{K_{B}T}{e}=0.025V$ is the thermal voltage and n is defined as an effective ideality factor that indicates the logarithmic scaling of the photo‐induced spectral shift with vis intensity. The dashed colored curves in Figure 4b,c, are the fit determined by the drift diffusion model. The extracted ideality factors are $\left|n\right|$ = 2.75 for diamond‐ and $\left|n\right|$ = 0.61 for triangular‐shaped sectors. This significant difference of n between the two sectors types, may indicate distinct underlying mechanisms. Ideality factors are defined in a Schottky contact. Having in mind that a very thin conductive oxide layer forms on rubrene crystals [32], which can take the role as a “metallic” electrode, the ideality factors of the diamond sectors, exceeding the “ideal” situation ‐that charge carriers stay separated (n≈1)‐ may be framed as follows: any light or electric field dependent photocurrents or trapping usually tend to increase ideality factors [33, 34]. Trap‐assisted (Shockley Read Hall) type of recombination (n = 2), we regard as unlikely because the photoemission is not stronger at borders or at domain boundaries. In our luminescence studies [19], we observed distinctly differing photon yield in the sectors, possibly involving loss of charge carriers due to recombination. This carrier leak could drive the ideality factors to values beyond 1. Potential reasons for sub‐unity ideality factors in general are three‐particle recombination (e.g., two majority carrier with one minority carrier) [35], surface / interface recombination [35, 36] and saturation of traps [37]. Note, that these are light ideality factors, which are more prone to reach sub‐unity values [36]. A small effect could arise in case temperature would rise beyond room temperature; this would artificially lower n. Additionally, for the more strongly excited and emitting triangular sectors, we cannot exclude space charge limited currents already being involved, which would not be covered by the drift‐diffusion model. The solid black curves in Figure 4b,c are the plots representing the sum of both equations with the fit parameters determined independently.

We attribute this light‐induced potential change to the formation of a photovoltage, as reported for rubrene [38, 39]. The observation of such a photovoltage implies the generation of mobile charge carriers. The typical depletion of charge carriers at a semiconductor surface is associated with an electric field and band bending, contributing to the surface potential. This field extends into the crystal according to the diffusion length of the relevant carriers. Upon illumination, the band bending is reduced, leading to a surface photovoltage that partially restores bulk‐like conditions. As noted above, rubrene molecular crystals are highly anisotropic, and so are their surface potentials and corresponding surface photovoltage contributions.

### 3.3 Surface Potential Investigations

**FIGURE 5:**
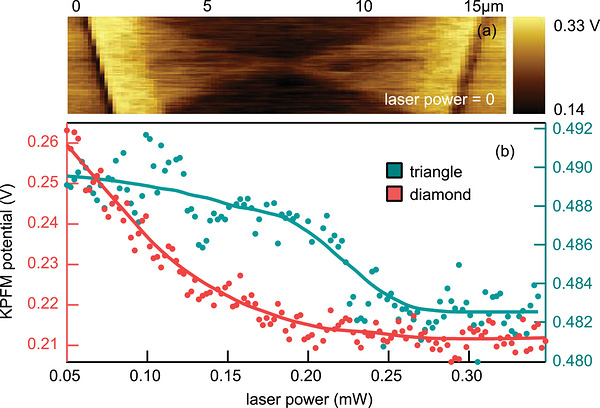
(a) KPFM map of a rubrene crystal showing the contrast of diamond‐ and triangular‐shaped zones, indicating different intrinsic work function of different zones. (b) KPFM potential of a rubrene microcrystal versus grazing illumination intensity, resolved for different sectors.

Regarding that the Surface potential of rubrene orthorhombic crystal is independent of the thickness, we repeatedly measured ≈ +300 mV for **c**‐oriented (**c**‐axis perpendicular to the crystal surface) and ≈ ‐150 mV for **b**‐oriented (**b**‐axis perpendicular to the surface) type, without illumination. Surface potential difference due to unit cell orientation is compatible with the common case that more densely packed surfaces exhibit a higher work function: both surface unit cells host two molecules, however the unit cell area of the **c**‐oriented phase is half the area of the **b**‐oriented, i.e., the **c**‐oriented has twice the density of species. However, such density dependent surface potential differences are of the order of 50 meV for molecules [40]. In our case, the surface potential differences are relatively larger (≈0.5V). As shown in Figure 5a, the crystal sectors (i.e., diamond‐ and triangular‐shaped) also exhibit contrast in the KPFM map, showing potential differences of about 100 mV, as variations in species density arise from the unit cell rotation within each domain. Having in mind, that tensile and compressive strain modulate the pinned Fermi level of rubrene by several 100 meV [41], together with the huge anisotropy in expansion coefficient along **a**‐ and **b**‐ lattice direction (of factor 5), and estimating the temperature during growth at room temperature, such large surface potential differences between crystal types (i.e., **c**‐ and **b**‐oriented) and sectors (i.e., diamond‐ and triangular‐shaped) may be attributed to residual stress upon cooling down the film after growth.

Figure 5b shows the KPFM potential of the diamond‐ and triangular‐shaped sectors of a **c**‐oriented rubrene microcrystal as a function of illumination intensity from a 532 nm laser applied at a grazing incidence of about 10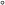. A drop in KPFM potential difference–corresponding to an increase in surface potential and charge carrier density (to diminish band bending)–is observed in both sectors. However, the reduction in KPFM potential relative to the laser intensity is significantly more pronounced in the diamond‐shaped sectors compared to the triangular‐shaped. The respective substrate remains unaffected. The different response to illumination in diamond‐ and triangular‐shaped sectors could be explained by different pinning of the Fermi level at the surface arising from the different defect concentration at the surface due to different unit cell rotation (see Figure 1).

In the overall picture, we explain the charging upon illumination as follows: Under UV excitation (6.2 eV), the one‐photon photoemission (1PPE) process liberates electrons similarly efficiently in the diamond‐shaped sectors and in the triangular ones. The photoemission leads to enhanced hole residuals near the surface, if the electron supplement from the substrate cannot fill up the holes as effective as the photoemission current. On the other hand, the most effective electron‐hole mobility direction is along the **a**‐axis for rubrene orthorhombic crystals [19]. Since the **a**‐axis has more out‐of‐plane component in triangular‐shaped sectors than in the diamond‐shaped, this leads to a different out‐of‐plane conductivity across the sectors, resulting in greater hole accumulation in the diamond sectors compared to the triangular ones. This accumulation increases over time characterized by a time constant. The photoemission yield gets reduced over time, photoelectrons lose kinetic energy and “apparent” binding energies get enhanced.

When the vis light source (3.1 eV) is applied, the generation of triplet excitons via singlet fission becomes the dominant process [24, 42, 43]. The long‐lived triplet excitons create a reservoir of holes, which can be filled by electrons from the substrate and subsequently transferred to the surface to neutralize the accumulated charge. This process occurs rapidly, effectively cancelling the charge pattern.

We measured the charging behavior in multiple samples (Figures S1–S5) of different types i.e., **c**‐ and **b**‐oriented. Figure S1 shows the same trend of spectral shift and charge pattern for diamond‐ and triangular‐shaped sectors in another **c**‐oriented crystal. Orthorhombic rubrene is known as one of the most anisotropic organic crystals, concerning conductivity, mobility [44, 45, 46, 47], among other. Since the charge landscape in rubrene zone‐sectored crystals is regarded as a direct consequence of the anisotropic out‐of‐plane conductivities across different zones, due to different out‐of‐plane **a**‐axis orientation, a **b**‐oriented crystal is expected to show no charge landscape resembling the zone‐sectoring pattern. In a **b**‐oriented zone‐sectored crystal, the **b**‐axis is oriented perpendicular to the crystal surface and the unit cell of diamond‐shaped sectors is rotated around the **a**‐axis [19]. The **a**‐axis is oriented equally in‐plane for both sectors, and there is no pronounced out‐of‐plane anisotropic conductivity (see Figure S2).

## 4 Conclusion

In this work, we used photoemission electron microscopy and spectroscopy to investigate the charge accumulation in zone‐sectored rubrene microcrystals under the influence of the external and internal photoemission process. The resulting charge patterns resemble the diamond‐ and triangular‐shaped sectors, revealing distinct charging behaviors in different zones. Notably, charge accumulation can be effectively suppressed by introducing an additional internal photoelectron excitation using illumination energies below the sample's work function, which is attributed to the generation of electron–hole pairs. The charge dynamics and energetics are described by a RC model and drift diffusion. The compatibility of the drift‐diffusion model with the observed energy shift in the high power regime implies an out of plane surface Schottky contact, as expected for a semiconducting sample. Our findings show that the zone‐sectored tabular rubrene single crystals consist of zones with different out‐of‐plane conductivities and open a route to control and design spatially resolved charge landscapes in organic semiconductor systems.

## Conflicts of Interest

The authors declare no conflicts of interest.

## Supporting information


**Supporting File**: advs76246‐sup‐0001‐SuppMat.pdf.

## Data Availability

The data that support the findings of this paper are openly available on the following address: $doi.org/10.18453/rosdok_{i}d00005529$
