# Supplementary material for: Dual‐Wavelength Control of Charge Accumulation in Rubrene Microcrystals with Anisotropic Conductivity
Source: Adv Sci (Weinh). 2026 Jun 23;13(52):e76246. doi: 10.1002/advs.76246 (PMC13336718; doi:10.1002/advs.76246)
Supplement: Supplementary file 1 [file ADVS-13-e76246-s001.pdf]

Supplementary information

Dual-wavelength control of charge accumulation in rubrene microcrystals with anisotropic conductivity

Moha Naeimi[1,2], Ingo Barke[1,2] and Sylvia Speller[1,2]

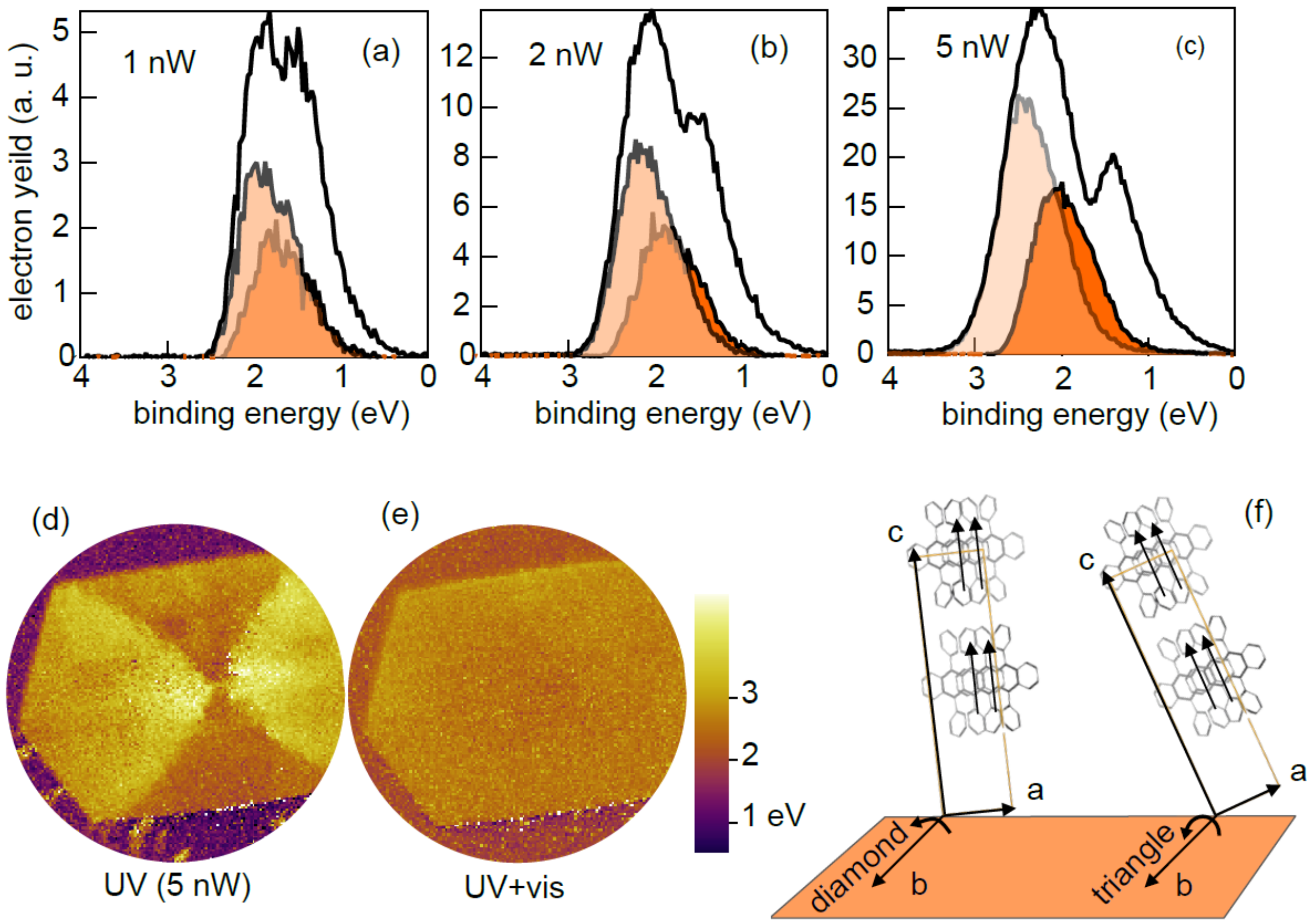

Figure 1 (a, b and c) Electron spectra from a different c-oriented sample excited 6.2 eV (UV) laser with different intensities, resolved for diamond- (shown as light fill-colored curves) and triangular-shaped (shown as thick fill-colored curves) sectors. The solid black curves are the overall electron spectra. The electron spectra from the diamond-shaped sectors shift toward higher "apparent" binding energies, due to photo-hole accumulation. This accumulation is due to low out-of-plane conductivity in diamond sectors compared to triangular sectors. Note that the difference between the sample in Figure 1 and the sample analyzed in the main manuscript is the swapped electron yield between diamond- and triangular-shaped sectors. While in the sample analyzed in the main manuscript, the electron yield in triangular sectors is larger than in diamond sectors, in the sample in the present figure, the diamond sectors exhibit more electron yield than the triangular sectors. This difference is due to different orientation between the emission dipole moment and light polarization among different samples. (d and f) Time-of-flight maps of the c-oriented sample, excited with UV-vis and UV-only lights, respectively. The 1PPE charge landscape reminiscent of the "hourglass" signature see [1], when the sample is illuminated with UV light carrying photons with energies equal to 6.2 eV. This charge pattern is cancelled out with a secondary illumination with photon energies equal to 3.1 eV. Field of view: 58 µm

[1] Institute of physics, University of Rostock, Rostock, Germany

[2] Department life, light & matter, University of Rostock, Germany

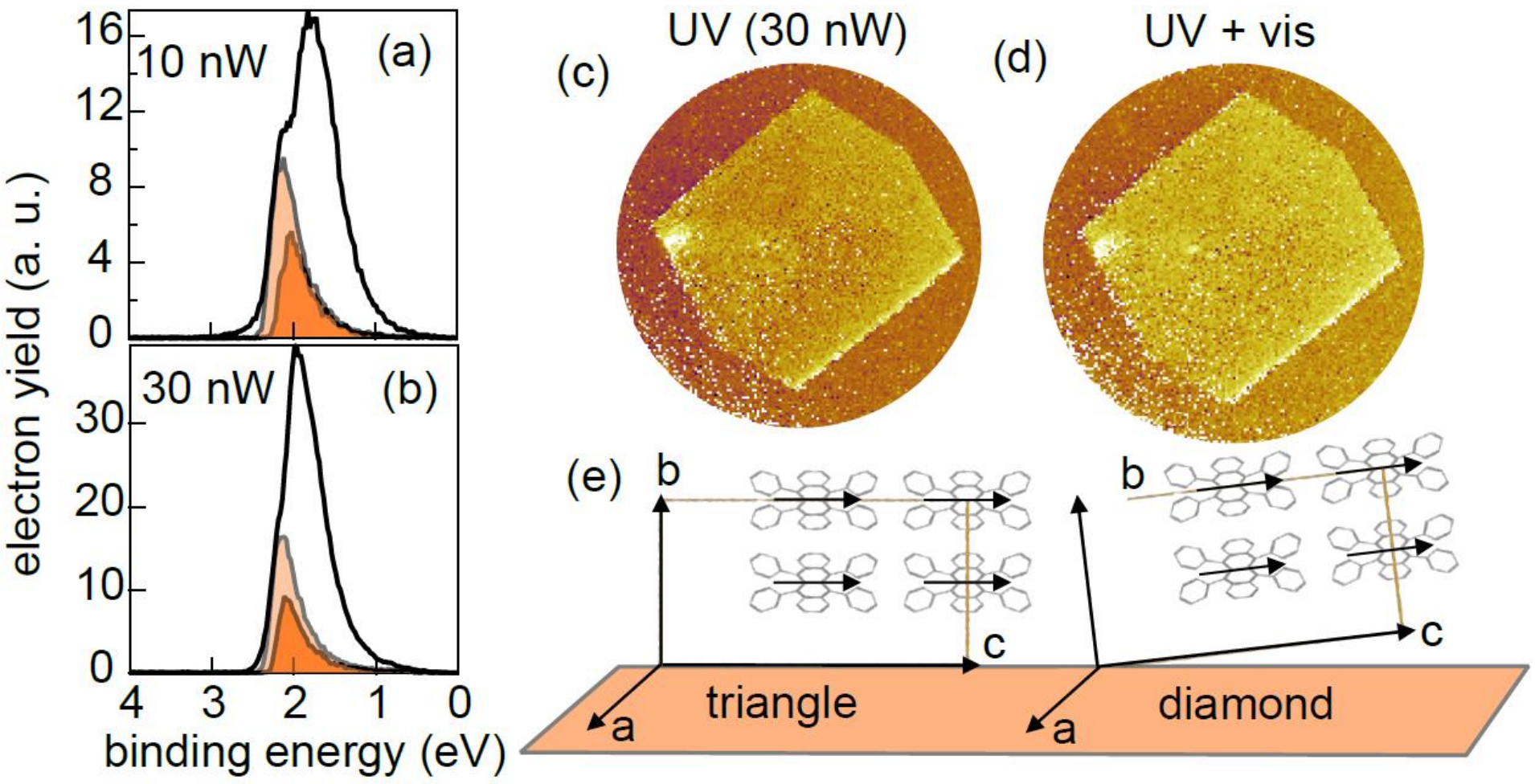

Figure 2 (a and b) Electron spectra of a b-oriented sample, resolved for diamond- (shown as light fill-colored curves) and triangular-shaped (shown as thick fill-colored curves) sectors, excited with a 6.2 eV (UV) laser, with two different illumination intensities. The solid black curves are the overall electron spectra. (c) Time-of-flight map of an a-oriented sample, excited with a 6.2 eV (UV) laser, showing a faint charge pattern despite intense illumination, as opposed to the sample shown in figure 1 and the sample analyzed in the main manuscript. (d) Time-of-flight map of the same sample illuminated with UV-vis lights. The charge pattern cancels out with the vis illumination. The rotation of the unit cell in different sectors is around the a-axis. Although a very subtle rotation around the b-axis is also present, as we discussed in [1]. Therefore, the out-of-plane conductivity is roughly the same for different sectors. Also, due to weak match of the polarization of the excitation light to the transition dipole moment, the photoemission is very subtle roughly the same the substrate (see Figure 3 sample #4). Field of view: 58 µm

---

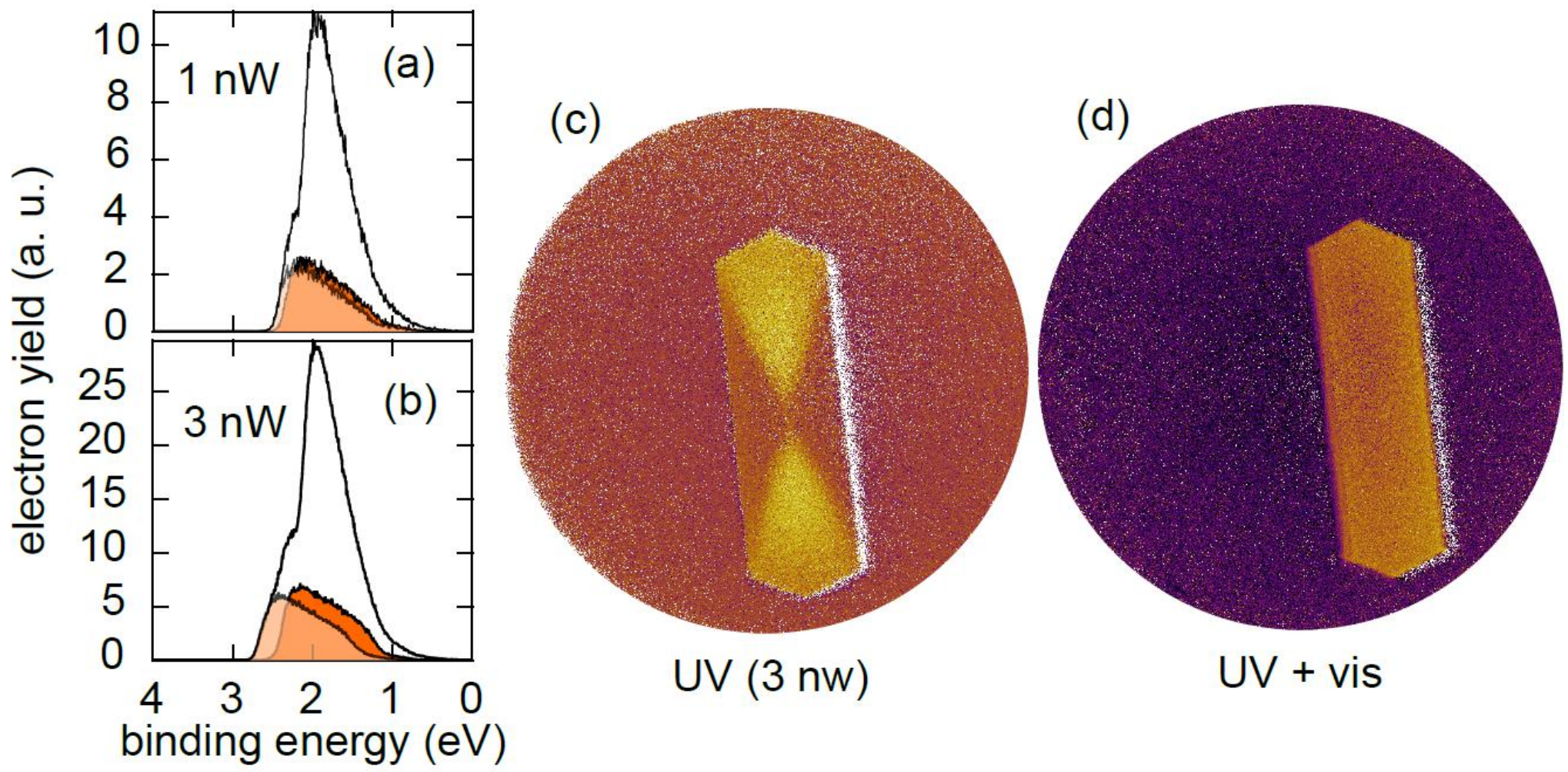

Figure 3 (a and b) Electron spectra of another c-oriented sample, resolved for diamond- (shown as light fill-colored curves) and triangular-shaped (shown as thick fill-colored curves) sectors, excited with a 6.2 eV (UV) laser, with two different illumination intensities. The solid black curves are the overall electron spectra. (c) Time-of-flight map of a c-oriented sample, excited with a 6.2 eV (UV) laser, showing charge landscape resembling the zone-sectoring pattern similar to the sample shown in figure 1 and the sample analyzed in the main manuscript. (d) Time-of-flight map of the same sample illuminated with UV-vis lights. The charge pattern cancels out with the vis illumination. Field of view: 58 µm

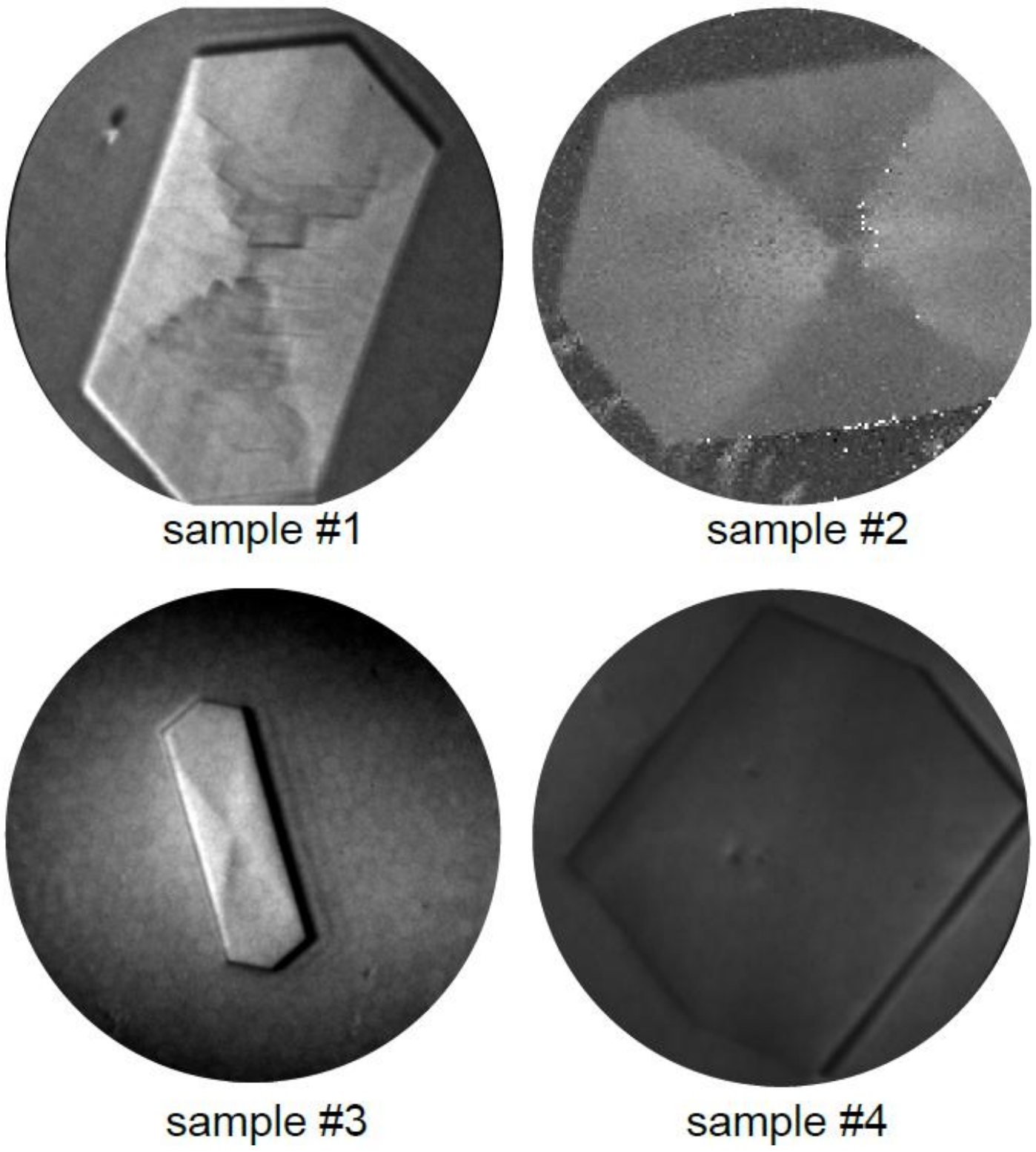

Figure 4 Series of CCD-PEEM images of different samples, analyzed in the present paper, excited with a UV laser, carrying photons with energies equal to 6.2 eV. Samples 1 (main manuscript) to 3 are all c-oriented, i.e. the c-axis of the orthorhombic unit cell is perpendicular to surface. In this crystal type, the unit cell is oriented differently in different sectors, around the b-axis, forming different out-of-plane a-axis and hence, different conductivities among diamond- and triangular-shaped sectors. Sample 4 is a b-oriented crystal [1] i.e. the b-axis is perpendicular to the surface. In this crystal type, the unit cell is oriented differently around the a-axis in different sectors. In this configuration, the out-of-plane a-axis is absent in both crystal sectors. Field of view: 58 µm

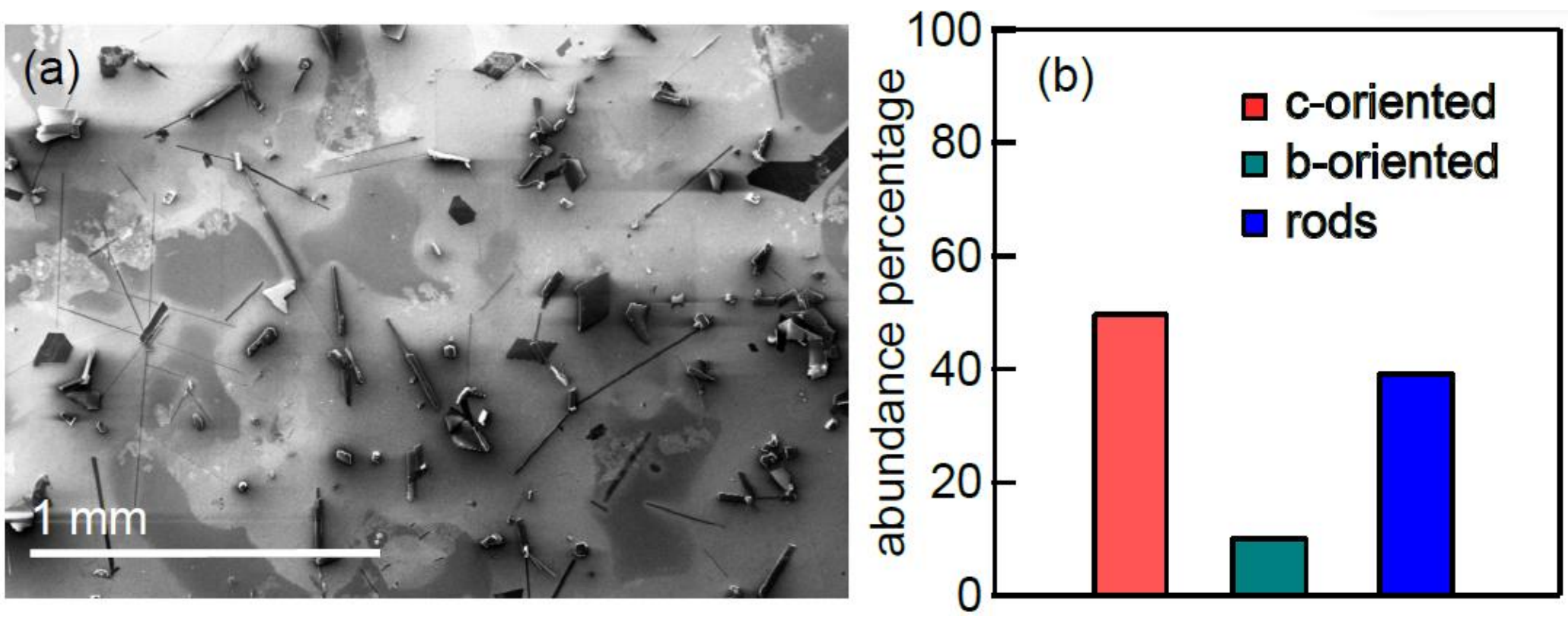

Figure 5 (a) SEM overview image of the prepared sample of rubrene micro-crystals with different morphologies (see [1]) (b) Abundance percentage of different morphologies.

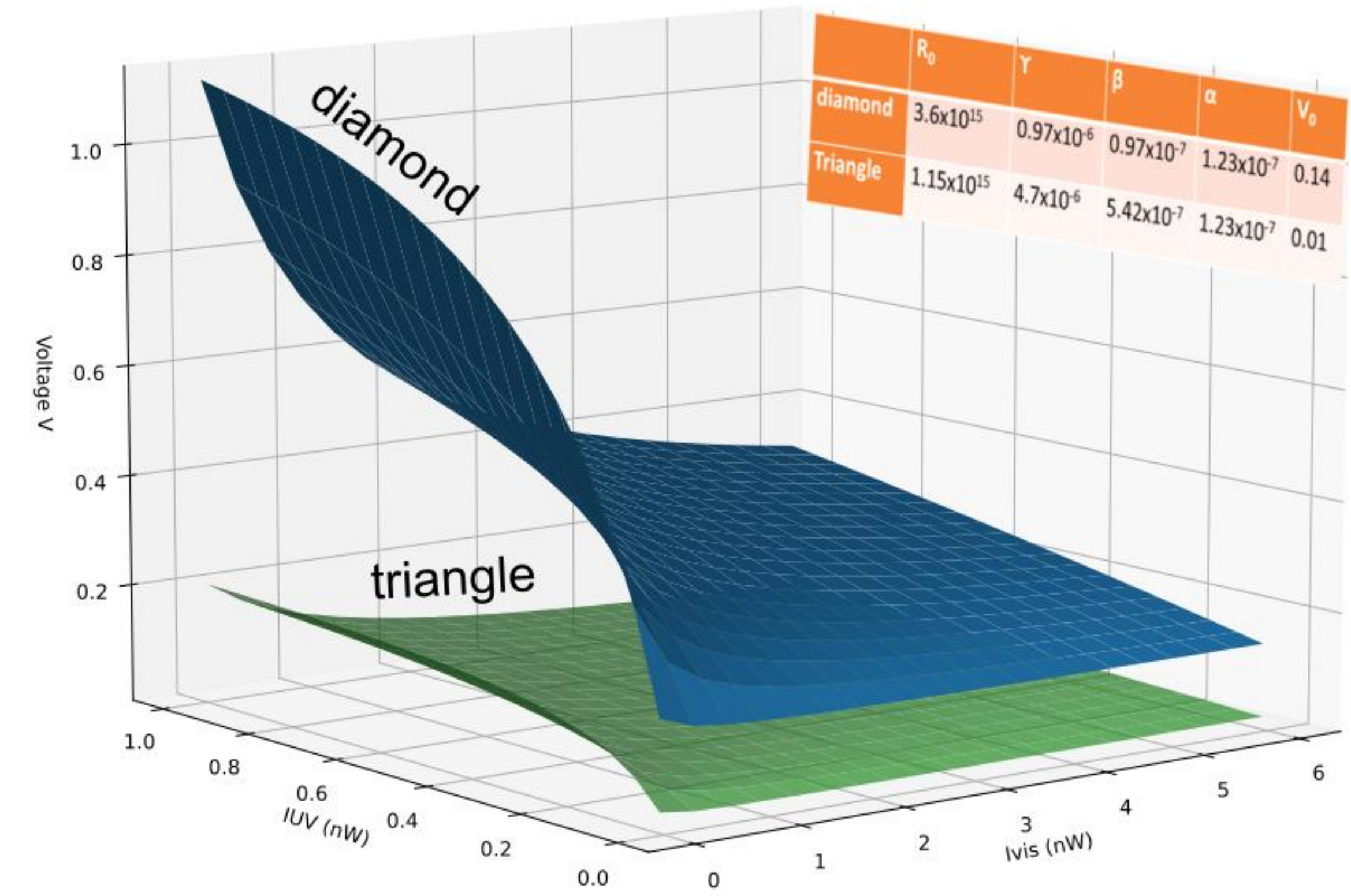

Figure 6 UV-vis control of charge landscape in zone-sectored crystals. 3D Simulation of the equation 3 in the manuscript, with the parameters obtained for sample #1.

References:

[1] M. Naeimi, T. Völzer, R. Lange, K. Oldenburg, S. Lochbrunner, I. Barke, and S. Speller, *Zone-sectored organic crystals with spatially resolved exciton dynamics*, Advanced Optical Materials 10.1002/adom.202502744 (2025).
